# Effectiveness of massage therapy on anxiety and depression of COVID-19 convalescent: A protocol for systematic review and meta-analysis

**DOI:** 10.1097/MD.0000000000031650

**Published:** 2022-11-25

**Authors:** Pan Huichai, Zhou Kelin, Dong Shuo, Liu Ting, Wang Jing, He Zhongchen, Pan Fang

**Affiliations:** a Beijing Hepingli Hospital, Beijing, China; b Beijing Dongcheng District Community Health Service Management Center, Beijing, China.

**Keywords:** 19, complementary and alternative medicine, massage therapy, patients recovering from COVID, protocol

## Abstract

Novel coronavirus pneumonia was first detected in Wuhan, China, in late December 2019. Its widespread infectivity and strong pathogenicity has posed a great threat to public health, seriously affecting social production and life. The disease caused by this virus has been officially named COVID-19 (coronavirus disease 2019) by the World Health Organization (WHO). Tuina (massage) therapy is 1 of the widely employed complementary and alternative medicine interventions in the world. As a useful therapy implemented on human’s skin, muscles and joints, tuina (massage) has unique advantages in the field of medicine. This systematic review and meta-analysis will summarize the current evidence of tuina (massage) used as an intervention for COVID-19.

**Methods:**

We will search the following electronic databases for randomized controlled trials to evaluate the effectiveness and safety of massage therapy in treating all patients recovering from COVID-19: Wanfang and Pubmed Database, china national knowledge infrastructure database, cochrane central register of controlled trials, cumulative index of nursing and allied health literature and excerpta medica database. Each database will be searched from inception to October 2022. The entire process will include study selection, data extraction, risk of bias assessment and meta-analyses.

**Results:**

This proposed study will evaluate the effectiveness and safety of massage therapy for patients recovering from COVID-19.

**Conclusions:**

This proposed systematic review will evaluate the existing evidence on the effectiveness and safety of massage therapy for patients recovering from COVID-19.

## 1. Introduction

Novel coronavirus pneumonia was first detected in Wuhan, China, in late December 2019.^[1]^ Its widespread infectivity and strong pathogenicity has posed a great threat to public health, seriously affecting social production and life. The disease caused by this virus has been officially named COVID-19 (coronavirus disease 2019) by the World Health Organization (WHO).^[2]^ Tuina (massage) therapy is 1 of the widely employed complementary and alternative medicine interventions in the world.^[3]^ As a useful therapy implemented on human’s skin, muscles and joints, tuina (massage) has unique advantages in the field of medicine.^[4]^ This systematic review and meta-analysis will summarize the current evidence of tuina (massage) used as an intervention for COVID-19.

This review aims to systematically review all randomized controlled trials (RCTs) to assess the effectiveness and safety of massage treatment for patients recovering from COVID-19.

## 2. Materials and methods

This systematic review protocol has been registered on PROSPERO on March 29, 2021 (Registration number: CRD42020196674). The protocol follows the Cochrane Handbook for Systematic Reviews of Interventions and the preferred reporting items for systematic reviews and meta-analysis protocol statement guidelines.^[5]^ We will describe the changes in our full review if needed. The protocol is funded through a protocol registry. This protocol is available from: https://www.crd.york.ac.uk/prospero/display_record.php?ID=CRD42020196674.

## 3. Inclusion criteria for study selection

### 3.1. Type of studies

This review will include clinical RCTs of clinical massage therapy for patients recovering from COVID-19 without any language or publication status restrictions. Non-RCTs, quasi-RCTs, case series, case reports, crossover studies, uncontrolled trials, and laboratory studies will not be included.

### 3.2. Type of participants

All patients recovering from COVID-19. All included participants in this review regardless of their age, race and gender.

### 3.3. Type of interventions

Interventions will include any type of clinically performed massage for improvement of pulmonary function in COVID-19. This will include Chinese Massage, Japanese Massage, Thai Massage, Swedish Massage, Tuina, Shiatsu, Remedial Massage, General Massage, Acupressure, Reflexology, Manual Lymphatic Drainage. Studies combined with other interventions such as acupuncture, herbal medicines, qigong and yoga will be considered for exclusion.

Control: no intervention, treatments other than massage (e.g. usual or standard care, placebo, wait-list controls).

### 3.4. Type of outcome measures

#### 3.4.1. Main outcome(s).

The influence of massage on the Muscle function and quality of life in convalescent patients. Comparison of improvement of main symptoms such as Depression Rating Scale and Anxiety Rating Scale, for example: Self-rating depression scale, Self-Rating Anxiety Scale, Hamilton Depression Scale; compare the differences in the scores of the World Health Organization’s Quality of Life Rating Scale (WHOQOL-100) before and after treatment.

#### 3.4.2. Additional outcome(s).

Accompanying symptoms (such as myalgia, expectoration, stuffiness, runny nose, pharyngalgia, anhelation, chest distress, dyspnea, crackles, headache, nausea, vomiting, anorexia, diarrhea) disappear rate, negative COVID-19 results rate on 2 consecutive occasions (not on the same day), CT image improvement, average hospitalization time, occurrence rate of common type to severe form, clinical cure rate, and mortality.

## 4. Search methods for the identification of studies

### 4.1. Electronic searches

We will search the following electronic bibliographic databases for relevant trials:

- China National Knowledge Infrastructure Database, from 1979 to present;
- Wanfang Database (from 1990 to present);
- Pubmed Database (from 2000 to present);
- Cochrane Central Register of Controlled Trials, from 2000 to present;
- Cumulative Index of Nursing and Allied Health Literature, from 1937 to present;
- Excerpta Medica database, from 1947 to present;
- Ovid MEDLINE ALL (Ovid Medical Literature Analysis and Retrieval System Online, from 1946 to present).

In addition, Clinical trial registries, like the Chinese Clinical Trial Registry (ChiCTR), the Netherlands National Trial Register (NTR) and ClinicalTrials.gov, will be searched for ongoing trials with unpublished data.

There will be no language restrictions.

### 4.2. Data collection and analysis

#### 4.2.1. Study identification.

We will use EndNote X9 software to manage the records of searched electronic databases. The initial selection will involve scanning of the titles and abstracts of the retrieved studies. The full text of relevant studies will then be reviewed for study inclusion, in accordance with the inclusion criteria, by 2 authors (PHC and ZKL). Potentially relevant articles will be reviewed independently by 2 authors to determine if they meet the prespecified criteria. Any disagreement between authors will be resolved by consensus with a third author. The study selection procedure will follow and be recorded in the PRISMA flow chart. All the evidence will be assessed by the grading of recommendations assessment, development and evaluation.

#### 4.2.2. Data extraction and management.

According to the inclusion criteria, a standard data collection form will be made before data extraction. The following data will be extracted by 2 authors (DS and ZKL):

- *General information*: Research identification, publication year, the title of the study, first author.
- *Study methods*: study design, sample size, randomization method, allocation concealment, blinding, incomplete report or selecting report, other sources of bias.
- *Participants*: Inclusion and exclusion criteria.
- *Intervention*: motion details, treatment duration, and frequency.
- *Control*: Type of control methods, motion details, treatment duration, and frequency.
- *Outcomes*: Included outcome measures.

#### 4.2.3. Risk of bias assessment.

The risk of bias in included studies will be assessed independently by 2 reviewers (DS and ZKL) using the Cochrane Risk of Bias Tool, with any disagreements resolved by consensus or by discussion with a third reviewer. All judgments will be fully described, and the conclusions will be presented in the Risk of Bias figures and will be incorporated into the interpretation of review findings, by means of sensitivity analysis. The risk of bias of each domain will be graded as adequate, unclear, or inadequate. We intend to use the concealment of allocation grading in investigation of any heterogeneity and in sensitivity analysis. Other aspects of study quality including the extent of blinding (if appropriate), losses to follow up, noncompliance, whether the outcome assessment was standardized, and whether an intention to treat analysis was undertaken, will be presented in the risk of bias table describing the included studies and will provide a context for discussing the reliability of the results.

#### 4.2.4. Data analysis.

We will use Stata Software [Computer program] (Version 15.1) to process the meta-analysis. Weighted mean difference will be used for continuous variable data, and the combined statistical effects of these 2 are combined. The *χ*^2^ test will be adopted to analyze whether there is heterogeneity in each of the included research questions. *I*^2^ > 50% is a criterion for significant judgment. The fixed effect model is adopted if *I*^2^ ≤ 50%, which is considered to have homogeneity between the studies. The random effect model is adopted if *I*^2^ > 50%, which is considered to have heterogeneity among the studies. The effect size is expressed as 95% confidence interval, and *P* < .05 is considered to be statistically significant.

***Sensitivity analyses:*** heterogeneity may be due to the presence of 1 or more outlier studies with results that conflict with the rest of the studies. We will perform sensitivity analyses excluding outlier studies. In addition, we plan to perform sensitivity analysis to explore the influence of trial quality on effect estimates. The quality components of methodology include adequacy of generation of allocation sequence, concealment of allocation, and the use of intention-to-treat analysis.

***Meta-regression analyses:*** if data permits, we will perform the meta-regression analyses.

#### 4.2.5. Publication bias.

If sufficient number of trials (more than 10 trials) are found, we will generate funnel plots (effect size against standard error) to investigate publication bias.

#### 4.2.6. Ethics and dissemination.

The data used in this systematic review will be collected from published studies. Based on this, the study does not require ethical approval.

## Acknowledgments

The authors would like to thank all the researchers in our working group.

## Author contributions

**Methodology:** ZKL.

**Resources:** ZKL and DS.

**Data curation:** PHC and HZC.

**Funding acquisition:** PF.

**Software:** ZKL and DS.

**Supervision:** LT and WJ.

**Writing ‐ original draft:** PHC, ZKL, DS, LT and WJ.

**Writing ‐ review & editing:** PHC, ZKL, DS, LT and WJ.
